# Latent profile analysis of alexithymia and its influencing factors in patients undergoing total knee arthroplasty

**DOI:** 10.3389/fpubh.2026.1919123

**Published:** 2026-08-12

**Authors:** Fengdi Zhang, Haoyue Gao, Xun Li, Yuling Ma, Yajie Deng, Yingxue Mei

**Affiliations:** 1 School of Nursing, Ningxia Medical University, Yinchuan, China; 2 Medical Education Administration Division, People's Hospital of Ningxia Hui Autonomous Region, Ningxia Medical University, Yinchuan, China

**Keywords:** alexithymia, latent profile analysis, nursing, pain intensity, social support, total knee arthroplasty

## Abstract

**Background:**

Alexithymia, characterized by difficulty identifying and describing emotions and a tendency toward externally oriented thinking, is common among people living with chronic pain and may compromise perioperative communication and rehabilitation engagement. However, previous studies have largely adopted variable-centered approaches, which may overlook clinically meaningful heterogeneity in alexithymia among patients undergoing TKA.

**Objective:**

To identify latent alexithymia profiles using latent profile analysis (LPA) of alexithymia among patients undergoing TKA and to determine demographic, clinical, and psychosocial factors associated with profile membership.

**Methods:**

A cross-sectional survey was conducted among 467 patients scheduled for TKA at a tertiary hospital in Ningxia, China, between July 2025 and April 2026. Participants completed a general information questionnaire, the Chinese version of the 20-item Toronto Alexithymia Scale (TAS-20), the Numerical Rating Scale for pain (NRS), the Hospital Anxiety and Depression Scale (HADS), and the Perceived Social Support Scale (PSSS). LPA was conducted using the three TAS-20 subscale scores (difficulty identifying feelings, difficulty describing feelings, and externally oriented thinking) as profile indicators. Multinomial logistic regression was used to identify factors associated with profile membership.

**Results:**

The mean TAS-20 score was 57.02 ± 5.76, indicating moderate alexithymic traits. A three-profile solution was identified: emotionally competent (*n* = 106, 22.70%), emotionally expressive difficulty (*n* = 197, 42.18%), and emotional-cognitive deficit (*n* = 164, 35.12%). Educational level, monthly income, pain duration, pain intensity, anxiety, depression, and perceived social support were associated with profile membership in univariable analyses (all *p* < 0.05). In multivariable multinomial logistic regression, higher perceived social support was associated with lower odds of membership in both the emotionally expressive difficulty profile (OR = 0.883, 95% CI: 0.793–0.983) and the emotional-cognitive deficit profile (OR = 0.767, 95% CI: 0.673–0.873). The emotional-cognitive deficit profile was additionally associated with higher anxiety scores (OR = 1.363, 95% CI: 1.124–1.652) and was associated with a broader range of adverse socioeconomic and psychological factors. Than the emotionally expressive difficulty profile (e.g., pain intensity: OR = 8.517 vs. 2.356; monthly income < 2,000 RMB: OR = 10.549 vs. 3.592).

**Conclusion:**

Alexithymia in patients undergoing TKA comprises three clinically distinguishable profiles. Profile membership is closely related to socioeconomic status, pain burden, psychological distress, and social support.

**Implications for nursing:**

Routine preoperative TAS-20 screening could facilitate early identification of patients at risk for alexithymia-related communication barriers. Profile-specific interventions should combine tailored communication strategies, pain management, psychological support, and family-inclusive social support, particularly for patients with emotional-cognitive deficits.

## 1 Introduction

Total knee arthroplasty (TKA) is an established surgical treatment for end-stage knee osteoarthritis (KOA) and is effective in relieving pain, correcting deformity, restoring function, and improving quality of life (1, 2). Despite favorable surgical outcomes for most patients, a substantial proportion of patients remain dissatisfied after TKA, and psychological factors are increasingly recognized as important determinants of postoperative recovery and patient-reported outcomes (3, 4).

Alexithymia refers to difficulty identifying feelings, difficulty describing feelings, and a cognitive style characterized by externally oriented thinking (5). Unlike anxiety or depression, which describe emotional states, alexithymia reflects impaired emotional awareness and communication. It affects approximately 10–13% of the general population (6, 7), but is more common in chronic pain populations (8, 9). Patients awaiting TKA often experience persistent joint pain, restricted mobility, and uncertainty regarding postoperative recovery, which may increase psychological vulnerability and may be accompanied by alexithymic traits (10).

Alexithymia is clinically relevant in the perioperative TKA setting because successful rehabilitation requires accurate symptom reporting, active participation in exercise, and timely communication with health professionals. Patients with alexithymia may have difficulty distinguishing physical discomfort from emotional distress, expressing rehabilitation-related concerns, or requesting support (11). Longitudinal evidence in chronic pain suggests that alexithymia and depression can impair treatment adherence and recovery (12). More recent evidence also indicates that alexithymia may moderate the relationship between pain intensity and psychological dependence on pain medication (13). These findings highlight the potential value of assessing alexithymia as part of perioperative pain management.

Most available studies have examined alexithymia using total scale scores, which assumes that patients with similar total scores share comparable emotional processing patterns. This variable-centered approach may obscure clinically important heterogeneity (14). Latent profile analysis (LPA) is a person-centered method that classifies individuals into unobserved subgroups according to similar response patterns on observed indicators (15). LPA can therefore reveal distinct clinical phenotypes and provide a basis for more precise nursing assessment and intervention.

To our knowledge, no study has used LPA to characterize alexithymia profiles among patients undergoing TKA. This study aimed to (1) identify latent profiles of alexithymia among patients scheduled for TKA and (2) examine demographic, clinical, and psychosocial factors associated with profile membership. The findings may inform profile-specific perioperative nursing strategies, including individualized assessment and intervention, pain management, psychological support, and rehabilitation engagement.

## 2 Methods

### 2.1 Study design and participants

This cross-sectional study used convenience sampling to recruit patients scheduled for TKA at the Department of Orthopedic Surgery of a tertiary grade-A hospital in Ningxia Hui Autonomous Region, China, between July 2025 and April 2026. The inclusion criteria were as follows: (a) diagnosis of knee osteoarthritis (KOA) according to the Osteoarthritis Diagnosis and Treatment Guidelines (2018 edition) (16), and scheduled for unilateral total knee arthroplasty (including both primary and revision TKA); (b) aged ≥ 18 years; (c) able to read and communicate in Chinese; and (d) willing to provide written informed consent. The exclusion criteria were as follows: (a) diagnosed psychiatric disorder; (b) severe cardiac, cerebral, hepatic, renal, or other major organic disease; (c) current psychotherapy; or (d) inability to complete the questionnaire independently or with assistance.

Based on a pilot study conducted in June 2025 (*n* = 32; alexithymia was defined as a TAS-20 score > 60, with a prevalence of 31.25%), the required sample size was calculated using the cross-sectional survey formula n = Z_1 − *α*/2_^2^ × P(1 − P)/*δ*^2^, where α = 0.05, Z_1 − α/2_ = 1.96, *p* = 0.3125, and δ = 0.05 (17). The minimum required sample size was approximately 330. After allowing an additional 20% for possible incomplete or invalid questionnaires, the target sample size was 396. The final sample size also exceeded the minimum recommendations for latent profile analysis (> 300 participants) and multinomial logistic regression (18). The final sample of 467 participants exceeded this requirement. Pilot study participants were not included in the final analysis. The study was approved by the hospital ethics committee (Approval No. 2025-LL-036). All participants provided written informed consent before enrollment.

### 2.2 Measures

#### 2.2.1 General information questionnaire

A self-designed questionnaire was used to collect demographic and clinical data, including sex, age, marital status, educational level, residence, living arrangement, occupation, monthly personal income, primary source of income, medical insurance type, duration of knee pain, surgical site, surgical type, and number of comorbidities.

#### 2.2.2 Toronto alexithymia scale

The 20-item Toronto Alexithymia Scale (TAS-20) was developed by Bagby et al. in 1994 (27), and its Chinese version was translated and validated by Yi et al. (19). The TAS-20 contains three subscales: difficulty identifying feelings (7 items), difficulty describing feelings (5 items), and externally oriented thinking (8 items). Items are rated on a 5-point Likert scale from 1 (strongly disagree) to 5 (strongly agree), with five items reverse scored. Total scores range from 20 to 100, with higher scores indicating greater alexithymia. In this study, Cronbach’s alpha coefficients for the total scale and the three subscales were 0.83, 0.81, 0.78, and 0.75, respectively.

#### 2.2.3 Numerical rating scale

Pain intensity was assessed using the 11-point Numerical Rating Scale (NRS) (20), with scores ranging from 0 (no pain) to 10 (worst imaginable pain). Scores of 1–3, 4–6, and 7–10 indicate mild, moderate, and severe pain, respectively. The NRS is a simple, reliable, and valid instrument widely used for clinical pain assessment, including among older adults and patients with musculoskeletal disorders. In this study, participants were asked to rate their average knee pain intensity during the preceding 24 h on the day of the preoperative assessment, and the score was recorded based on the patient’s self-report.

#### 2.2.4 Hospital anxiety and depression scale

Symptoms of anxiety and depression were assessed using the Hospital Anxiety and Depression Scale (HADS), developed by Zigmond and Snaith (21). The scale consists of 14 items, including two 7-item subscales measuring anxiety (HADS-A) and depression (HADS-D). Each item is scored on a 4-point Likert scale (0–3), yielding subscale scores ranging from 0 to 21. Scores of 8 or higher on either subscale indicate clinically relevant anxiety or depressive symptoms (22). The Chinese version of the HADS has demonstrated satisfactory reliability and validity in various Chinese clinical populations, including orthopedic patients (23). In the present study, Cronbach’s alpha coefficients were 0.890 for the total scale, 0.820 for the anxiety subscale, and 0.807 for the depression subscale.

#### 2.2.5 Perceived social support scale

Perceived social support was measured using the Perceived Social Support Scale (PSSS), developed by Blumenthal et al. (24). The scale contains 12 items that assess perceived support from family, friends, and significant others. Items are rated on a 7-point Likert scale from 1 (very strongly disagree) to 7 (very strongly agree), with total scores ranging from 12 to 84. Higher scores indicate greater perceived social support. In this study, Cronbach’s alpha coefficient was 0.885.

### 2.3 Data collection

Data were collected by two graduate nursing students who received standardized training before the study to ensure consistency in questionnaire administration. Eligible participants were approached during their preoperative hospital stay, typically 1 day before surgery. After the study purpose and procedures had been explained, written informed consent was obtained from each participant.

Participants completed the questionnaires independently in a quiet room whenever possible. For those with limited literacy or visual impairment, the research assistants read each question aloud using standardized instructions and recorded participants’ responses without providing interpretation or guidance. Completed questionnaires were reviewed immediately for completeness and consistency, and any missing or ambiguous responses were clarified with participants on site.

### 2.4 Statistical analysis

Statistical analyses were performed using SPSS version 27.0 (IBM Corp., Armonk, NY, USA) and Mplus version 8.3 (Muthén & Muthén, Los Angeles, CA, USA). Before the formal analyses, the dataset was examined for completeness. No missing values were identified; therefore, neither data imputation nor casewise deletion was required.

Normally distributed continuous variables are presented as mean ± standard deviation (SD), non-normally distributed continuous variables as medians (P_25_, P_75_), and categorical variables as frequencies and percentages. Univariable analyses were conducted to compare sociodemographic and clinical characteristics across the identified latent profiles. The chi-square test was used for categorical variables. For continuous variables, one-way ANOVA was used for normally distributed variables, and the Kruskal–Wallis H test was used for non-normally distributed variables. The distributions of continuous variables were examined before group comparisons. Homogeneity of variance was assessed using Levene’s test. For variables satisfying the homogeneity-of-variance assumption, conventional one-way analysis of variance (ANOVA) followed by Bonferroni-adjusted pairwise comparisons was used. When the homogeneity-of-variance assumption was violated, Welch’s ANOVA followed by Games–Howell *post hoc* comparisons was applied. Effect sizes for overall between-profile differences were estimated using eta squared (η^2^).

Latent profile analysis (LPA) was conducted using the three TAS-20 subscale scores—Difficulty Identifying Feelings (DIF), Difficulty Describing Feelings (DDF), and Externally Oriented Thinking (EOT)—as profile indicators. Models specifying one to four latent profiles were estimated using maximum likelihood estimation with robust standard errors (MLR). Model fit was evaluated using the Akaike information criterion (AIC), Bayesian information criterion (BIC), sample-size-adjusted Bayesian information criterion (aBIC), entropy, the Lo–Mendell–Rubin adjusted likelihood ratio test (LMRT), and the bootstrap likelihood ratio test (BLRT). Lower values of AIC, BIC, and aBIC indicate better model fit, whereas entropy values ≥0.80 indicate satisfactory classification accuracy. Significant LMRT and BLRT results indicate that a model with k profiles provides a better fit than a model with k − 1 profiles (25). The optimal model was selected by jointly considering statistical fit indices, classification accuracy, profile size, parsimony, and clinical interpretability.

Before multinomial logistic regression, multicollinearity among the independent variables was assessed using variance inflation factors (VIFs). VIF values below 2.5 were considered to indicate the absence of substantial multicollinearity (26). Multinomial logistic regression was performed to examine predictors of latent profile membership, with the optimal latent profile classification as the dependent variable. Candidate predictors were selected based on clinical relevance, prior evidence, and univariable findings. Variables considered clinically important or potential confounders were retained in the multivariable model regardless of their univariable significance. Additional variables associated with profile membership at *p* < 0.05 in univariable analyses were also included. Results are presented as odds ratios (ORs) with 95% confidence intervals (CIs). All statistical tests were two-sided, and *p* < 0.05 was considered statistically significant.

## 3 Results

### 3.1 Participant characteristics

A total of 483 questionnaires were distributed, and 467 valid questionnaires were returned, yielding an effective response rate of 96.69%. Among the 467 patients scheduled to undergo total knee arthroplasty, the mean TAS-20 total score was 57.02 ± 5.76. The sample included 307 women (65.7%) and 160 men (34.3%), with a mean age of 67.4 ± 8.2 years (range, 38–89 years). Demographic and clinical characteristics across the three alexithymia profiles are shown in Table 1.

**Table 1:** Characteristics of patients undergoing total knee arthroplasty by alexithymia profile.

Characteristic	Category	Total (*N* = 467)	Emotionally competent (*N* = 106)	Emotionally expressive difficulty (*N* = 197)	Emotional-cognitive deficit (*N* = 164)	Test statistic	*P*
Gender	Female	307 (65.7)	62 (58.5)	129 (65.5)	116 (70.7)	χ^2^ = 4.294	0.117
	Male	160 (34.3)	44 (41.5)	68 (34.5)	48 (29.3)		
Age (years)	<=65	181 (38.8)	51 (48.1)	70 (35.5)	60 (36.6)	χ^2^ = 5.098	0.078
	>65	286 (61.2)	55 (51.9)	127 (64.5)	104 (63.4)		
Marital status	Unmarried/divorced/widowed	39 (8.4)	6 (5.7)	6 (3.0)	27 (16.5)	χ^2^ = 22.349	<0.001
	Married	428 (91.6)	100 (94.3)	191 (97.0)	137 (83.5)		
Education	Primary school or below	208 (44.5)	15 (14.2)	84 (42.6)	109 (66.5)	χ^2^ = 86.673	<0.001
	Junior high school	161 (34.5)	44 (41.5)	78 (39.6)	39 (23.8)		
	Senior high school or above	98 (21.0)	47 (44.3)	35 (17.8)	16 (9.7)		
Residence	Urban	274 (58.7)	73 (68.9)	108 (54.8)	93 (56.7)	χ^2^ = 6.010	0.050
	Rural	193 (41.3)	33 (31.1)	89 (45.2)	71 (43.3)		
Living arrangement	Living alone	20 (4.3)	7 (6.6)	5 (2.5)	8 (4.9)	χ^2^ = 2.998	0.223
	With family	447 (95.7)	99 (93.4)	192 (97.5)	156 (95.1)		
Occupation	Worker	47 (10.1)	7 (6.6)	22 (11.2)	18 (11.0)	χ^2^ = 27.741	<0.001
	Farmer	211 (45.2)	34 (32.1)	93 (47.2)	84 (51.2)		
	Civil servant/public institution	36 (7.7)	19 (17.9)	11 (5.6)	6 (3.7)		
	Retired	173 (37.0)	46 (43.4)	71 (36.0)	56 (34.1)		
Monthly income (RMB)	< 2,000	216 (46.3)	20 (18.9)	71 (36.0)	125 (76.2)	χ^2^ = 165.367	<0.001
	2,000–3,000	135 (28.9)	20 (18.9)	88 (44.7)	27 (16.5)		
	>3,000	116 (24.8)	66 (62.2)	38 (19.3)	12 (7.3)		
Primary source of income	Employment income	112 (24.0)	29 (27.4)	51 (25.9)	32 (19.5)	χ^2^ = 4.242	0.644
	Pension	116 (24.8)	27 (25.5)	50 (25.4)	39 (23.8)		
	Social security/subsistence allowance	163 (34.9)	36 (33.9)	64 (32.5)	63 (38.4)		
	None	76 (16.3)	14 (13.2)	32 (16.2)	30 (18.3)		
Pain duration (years)	<5	211 (45.2)	70 (66.0)	90 (45.7)	51 (31.1)	χ^2^ = 46.025	<0.001
	5–10	145 (31.0)	23 (21.7)	72 (36.5)	50 (30.5)		
	> 10	111 (23.8)	13 (12.3)	35 (17.8)	63 (38.4)		
Surgical site	Left knee	223 (47.8)	55 (51.9)	92 (46.7)	76 (46.3)	χ^2^ = 0.944	0.624
	Right knee	244 (52.2)	51 (48.1)	105 (53.3)	88 (53.7)		
Surgical type	Primary TKA	387 (82.9)	91 (85.8)	168 (85.3)	128 (78.0)	χ^2^ = 4.153	0.125
	Revision TKA	80 (17.1)	15 (14.2)	29 (14.7)	36 (22.0)		
Comorbidities	0	195 (41.8)	60 (56.6)	77 (39.1)	58 (35.4)	χ^2^ = 14.323	0.026
	1	180 (38.5)	34 (32.1)	78 (39.6)	68 (41.5)		
	≥2	92 (19.7)	12 (11.3)	42 (21.3)	38 (23.1)		
Pain intensity	Mean ± SD/median (P_25_, P_75_)	6.93 ± 1.18	6 (5–6)	7 (6–7)	8 (7–9)	H = 216.443	<0.001
HADS Anxiety	Mean ± SD/median (P_25_, P_75_)	8.36 ± 2.79	6 (4–8)	8 (7–9)	10 (9–12)	H = 118.826	<0.001
HADS Depression	Mean ± SD/median (P_25_, P_75_)	7.47 ± 3.04	5 (3.75–6)	7 (6–9)	9 (7–11.75)	H = 120.248	<0.001
Social support	Mean ± SD/median (P_25_, P_75_)	61.55 ± 4.46	65 (62.75–67.00)	62 (60.00–64.00)	59 (56.00–62.00)	H = 122.678	<0.001

HADS, Hospital Anxiety and Depression Scale; TKA, total knee arthroplasty. Categorical variables are shown as n (%). For pain intensity, HADS-Anxiety, HADS-Depression, and social support, the total column is shown as mean ± SD, and profile-specific values are shown as median (P_25_, P_75_). Test statistics are chi-square values for categorical variables and Kruskal–Wallis H values for non-normally distributed continuous variables.

**Figure 1:**
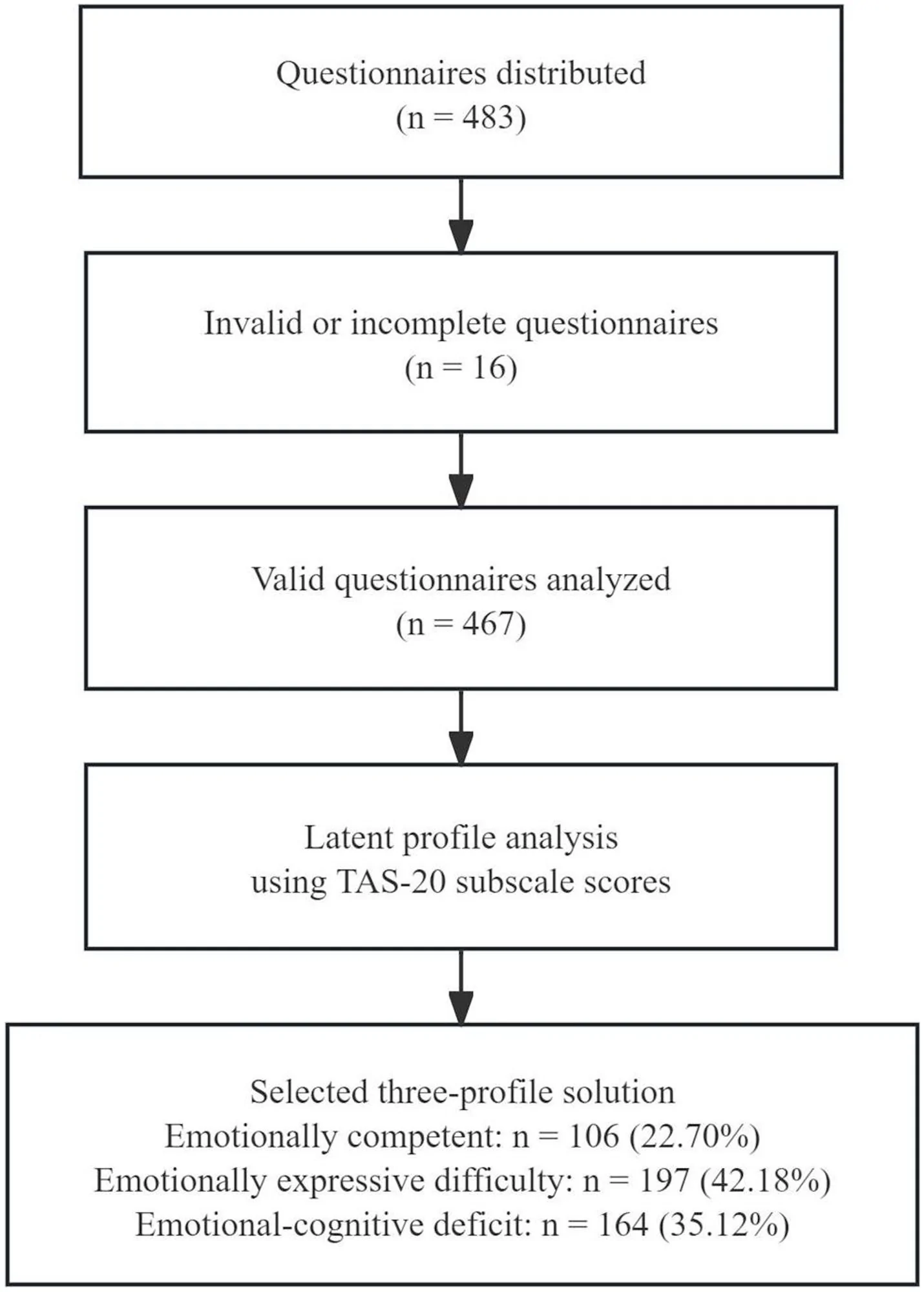
Participant flow and analytic sample. Of 483 distributed questionnaires, 467 valid responses were included in the final analysis and entered into latent profile analysis. The selected three-profile solution comprised the emotionally competent profile (*n* = 106, 22.70%), emotionally expressive difficulty profile (*n* = 197, 42.18%), and emotional-cognitive deficit profile (*n* = 164, 35.12%). TAS-20 = 20-item Toronto Alexithymia Scale.

The participant flow and analytic sample are summarized in Figure 1.

### 3.2 Comparison of characteristics across three latent profiles

The three profiles differed significantly in TAS-20 total score, pain intensity, HADS-A, HADS-D, and perceived social support (all *p* < 0.001; Table 2). TAS-20 total score, pain intensity, anxiety, and depression increased progressively across profiles from emotionally competent to emotionally expressive difficulty to emotional-cognitive deficit. Perceived social support showed the reverse pattern. All pairwise comparisons were significant (adjusted *p* < 0.001), with large effect sizes (η^2^ = 0.237–0.798).

**Table 2:** Comparison of characteristics across three latent profile (*N* = 467).

Variable	Profile 1: emotionally-competent (*N* = 106)	Profile 2: emotionally-expressive difficulty (*N* = 197)	Profile 3: emotional-cognitive deficit (*N* = 164)	*F*/Welch’s *F*	*P*	η^2^	*Post-hoc*
TAS-20 total score	49.42 ± 2.76	56.05 ± 2.24	63.09 ± 2.87	780.074	<0.001	0.798	1 < 2 < 3 (GH, all *P* < 0 0.001)
Pain intensity score	5.86 ± 0.92	6.69 ± 0.80	7.91 ± 0.92	191.602	<0.001	0.452	1 < 2 < 3 (Bonf., all *P* < 0.001)
HADS depression	5.10 ± 2.28	7.35 ± 2.45	9.16 ± 3.04	78.816	<0.001	0.248	1 < 2 < 3 (GH, all *p* < 0.001)
Social support score	64.58 ± 2.85	62.18 ± 3.41	58.84 ± 4.87	75.098	<0.001	0.244	1 > 2 > 3 (GH, all *P* < 0.001)
HADS anxiety	6.38 ± 2.68	8.07 ± 2.26	9.98 ± 2.49	72.185	<0.001	0.237	1 < 2 < 3 (Bonf., all *p* < 0.001)

Values are presented as mean ± standard deviation. TAS-20 = Toronto Alexithymia Scale-20. Post-hoc comparisons: GH = Games–Howell test; Bonf. = Bonferroni adjustment. All pairwise comparisons were significant (adjusted *p* < 0.001). 1 < 2 < 3 indicates Profile 1 < Profile 2 < Profile 3; 1 > 2 > 3 indicates Profile 1 > Profile 2 > Profile 3.

### 3.3 Identification of alexithymia profiles

The model-fit indices for the one- to four-profile solutions are presented in Table 3. AIC, BIC, and aBIC decreased as the number of profiles increased. The three-profile model had acceptable entropy (0.802), and both the LMRT and BLRT were statistically significant, indicating improved fit compared with the two-profile model. Although the four-profile model produced slightly lower information criteria, its entropy declined to 0.725 and the LMRT was not statistically significant (*p* = 0.051). The additional profile therefore did not provide sufficient improvement in classification quality to justify the increased model complexity. Considering statistical fit, classification accuracy, parsimony, profile size, and clinical interpretability, the three-profile solution was selected. Average posterior probabilities ranged from 0.900 to 0.930, indicating satisfactory classification accuracy.

**Table 3:** Fit indices for latent profile models (*N* = 467).

No. of profiles	LL	AIC	BIC	aBIC	Entropy	LMRT *P*	BLRT *P*	Class proportions
1	−3109.247	6230.494	6255.372	6236.329	–	–	–	1.000
2	−2912.200	5844.400	5885.864	5854.126	0.746	<0.001	<0.001	0.497/0.503
3	−2839.929	5707.858	5765.907	5721.474	0.802	<0.001	<0.001	0.227/0.422/0.351
4	−2829.186	5694.371	5769.005	5711.877	0.725	0.051	0.046	0.229/0.176/0.216/0.379

The selected model is the three-profile solution. LL, log-likelihood; AIC, Akaike information criterion; BIC, Bayesian information criterion; aBIC, adjusted Bayesian information criterion; LMRT, Lo–Mendell–Rubin adjusted likelihood ratio test; BLRT, bootstrap likelihood ratio test.

### 3.4 Characteristics of the three profiles

**Figure 2:**
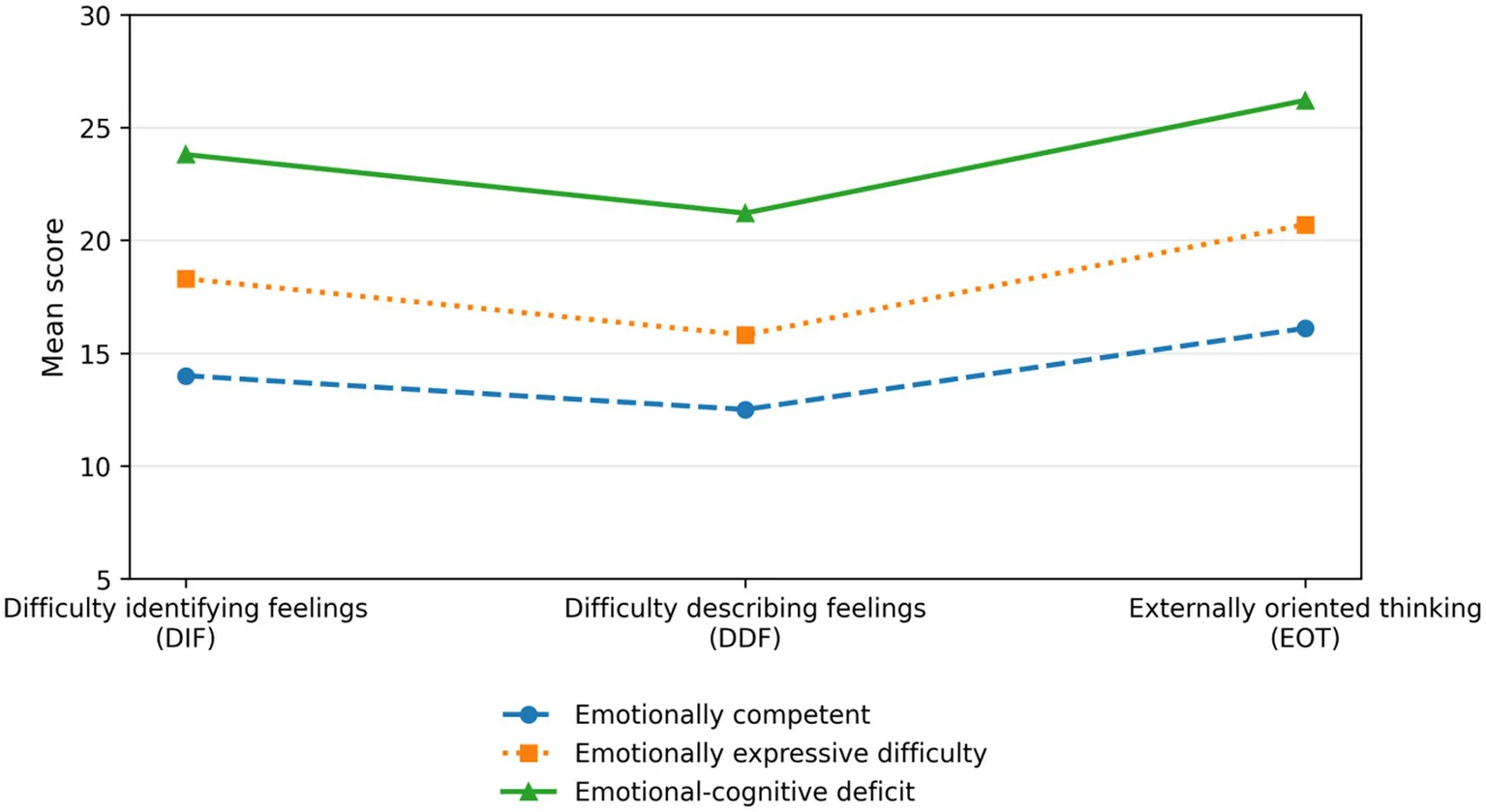
Latent profiles of alexithymia based on the three TAS-20 dimensions. Lines represent mean scores for difficulty identifying feelings, difficulty describing feelings, and externally oriented thinking. Higher scores indicate greater alexithymic traits. TAS-20 = 20-item Toronto Alexithymia Scale.

Based on the latent profile analysis results, alexithymia in TKA patients was classified into three profiles, as shown in Figure 2. The clinical significance and naming rationale for each profile are described below. Profile 1: Emotionally Competent (*n* = 106, 22.70%). This profile showed the lowest and relatively flat scores across all three TAS-20 dimensions, indicating intact emotional processing. This pattern aligns with the TAS-20 theoretical framework, in which low alexithymic traits represent preserved emotional capacities (27). Profile 2: Emotionally Expressive Difficulty (*n* = 197, 42.18%). This profile showed intermediate scores across all dimensions, with relatively higher difficulty describing feelings. Patients in this profile appear able to perceive emotions but experience marked difficulty in verbal expression. This pattern is consistent with the “affective awareness dysfunction” subtype, in which emotional perception is relatively preserved while expressive functioning is impaired (28). Profile 3: Emotional-Cognitive Deficit (*n* = 164, 35.12%). This profile showed the highest scores across all TAS-20 dimensions, indicating a broad impairment in emotional awareness, emotional expression, and internally oriented emotional processing. Patients in this profile exhibit deficits at the initial stage of emotional processing, tending to adopt an externally oriented cognitive style and experiencing substantial difficulty in recognizing their own emotions. This represents the most severe manifestation of alexithymia, consistent with the clinical concept of classic alexithymia (29). This profile represented the most extensive impairment across emotional processing dimensions and was therefore considered the highest alexithymia severity subgroup.

### 3.5 Common method bias test

Because all variables were measured using self-report questionnaires, common method bias (CMB) was assessed before hypothesis testing. Harman’s single-factor test showed that the first unrotated factor accounted for 12.19% of the total variance, which was substantially below the recommended threshold of 50% (30), suggesting that CMB was unlikely to be a serious concern. Competing confirmatory factor analyses further supported this conclusion (Table 4). The hypothesized three-factor model demonstrated substantially better fit than the single-factor model, with acceptable model fit according to conventional criteria. Specifically, compared with the single-factor model, the three-factor model showed lower χ^2^ values and improved fit indices (CFI = 0.959, TLI = 0.933, RMSEA = 0.069, and SRMR = 0.050), whereas the single-factor model showed poor fit (CFI = 0.831, TLI = 0.763, RMSEA = 0.130, and SRMR = 0.075). Taken together, these results indicate that common method bias was unlikely to have materially affected the findings of the present study.

**Table 4:** Common method bias test.

Model	χ2	df	CFI	TLI	RMSEA	SRMR
Single-factor model	178.031	20	0.831	0.763	0.130	0.075
Three-factor model	55.151	17	0.959	0.933	0.069	0.050

The three-factor model comprises alexithymia, anxiety/depression, and social support; the NRS (a single-item clinical measure) was not included. CFI, Comparative Fit Index; TLI, Tucker-Lewis Index; RMSEA, Root Mean Square Error of Approximation; SRMR, Standardized Root Mean Square Residual.

### 3.6 Factors associated with alexithymia profile membership

Before the multivariable multinomial logistic regression analysis, multicollinearity among the independent variables included in the model was assessed using variance inflation factors (VIFs). The VIF values ranged from 1.296 to 1.722, all of which were well below the conventional threshold of 2.5 (26), indicating no evidence of substantial multicollinearity. Univariable analyses showed significant differences across the three latent profiles in marital status, educational level, occupation, monthly income, type of medical insurance, duration of knee pain, number of comorbidities, pain intensity, HADS-Anxiety scores, HADS-Depression scores, and perceived social support (all *p* < 0.05; Table 1). Variables included in the multivariable logistic regression were selected based on clinical relevance and the univariable between-profile differences. Because patients undergoing primary and revision total knee arthroplasty may differ in pain experience, responses to surgical trauma, and psychological characteristics, surgical type (primary TKA vs. revision TKA) was considered a clinically relevant potential confounder and was also included in the multivariable multinomial logistic regression model. The final multivariable model included marital status, educational level, occupation, monthly income, type of medical insurance, duration of knee pain, surgical type, number of comorbidities, pain intensity, HADS-Anxiety scores, HADS-Depression scores, and perceived social support. Table 5 presents the results of the adjusted multinomial logistic regression analysis. After adjustment for all prespecified covariates, several factors remained significantly associated with latent profile membership. For clarity, Table 5 displays only variables with statistically significant associations, whereas nonsignificant covariates were retained in the fully adjusted model but are not presented.

**Table 5:** Multinomial logistic regression analysis of factors associated with alexithymia profile membership.

Variable	Reference	B	SE	Wald χ^2^	*P*	OR (95% CI)	VIF
Emotionally expressive difficulty vs. emotionally competent							
Constant	-	−0.348	4.079	0.007	0.932	-	-
Pain intensity	Continuous	0.857	0.217	15.617	<0.001	2.356(1.540–3.605)	1.583
HADS Depression	Continuous	0.188	0.078	5.885	0.015	1.207(1.037–1.405)	1.435
Social support	Continuous	−0.125	0.055	5.15	0.023	0.883 (0.793–0.983)	1.722
Education (primary school or below)	Senior high school or above	1.244	0.471	6.963	0.008	3.469 (1.377–8.738)	1.296
Income (< 2,000 RMB)	>3,000 RMB	1.279	0.442	8.381	0.004	3.592 (1.511–8.536)	1.514
Income (2,000–3,000 RMB)	>3,000 RMB	1.698	0.406	17.523	<0.001	5.464 (2.467–12.099)	1.514
Pain duration (5–10 years)	<5 years	0.689	0.34	4.123	0.042	1.992 (1.024–3.876)	1.321
Emotional-cognitive deficit vs. emotionally competent							
Constant	-	−6.523	4.939	1.744	0.187	-	-
Pain intensity	Continuous	2.142	0.28	58.666	<0.001	8.517 (4.923–14.734)	1.583
HADS Anxiety	Continuous	0.31	0.098	9.939	0.002	1.363 (1.124–1.652)	1.435
HADS Depression	Continuous	0.207	0.096	4.659	0.031	1.230 (1.019–1.485)	1.435
Social support	Continuous	−0.266	0.066	16.114	<0.001	0.767 (0.673–0.873)	1.722
Education (primary school or below)	Senior high school or above	2.028	0.647	9.815	0.002	7.597 (2.136–27.014)	1.296
Income (< 2,000 RMB)	>3,000 RMB	2.356	0.649	13.197	<0.001	10.549 (2.959–37.606)	1.514
Pain duration (> 10 years)	<5 years	1.065	0.446	5.717	0.017	2.901 (1.212–6.947)	1.321
Pain duration (5–10 years)	<5 years	0.892	0.389	5.257	0.022	2.439 (1.138–5.226)	1.321

Reference group, emotionally competent profile; CI, confidence interval; OR, odds ratio.

The multivariable multinomial logistic regression analysis is shown in Table 5. Both the emotionally expressive difficulty profile and the emotional-cognitive deficit profile differed from the emotionally competent profile in several respects. Pain intensity was markedly elevated in both alexithymia-related profiles, with a substantially larger effect observed for the emotional-cognitive deficit profile. Lower educational attainment and reduced perceived social support were common risk factors for membership in both profiles. The two alexithymia-related profiles also exhibited distinct characteristics. The emotionally expressive difficulty profile was specifically associated with intermediate pain duration (5–10 years) and elevated depressive symptoms, whereas the emotional-cognitive deficit profile was additionally characterized by more prolonged pain duration, was characterized by lower income level and higher anxiety symptoms (monthly income < 2,000 RMB). Notably, the magnitude of association with pain intensity was approximately 3.6-fold greater for the emotional-cognitive deficit profile than for the emotionally expressive difficulty profile.

**Figure 3:**
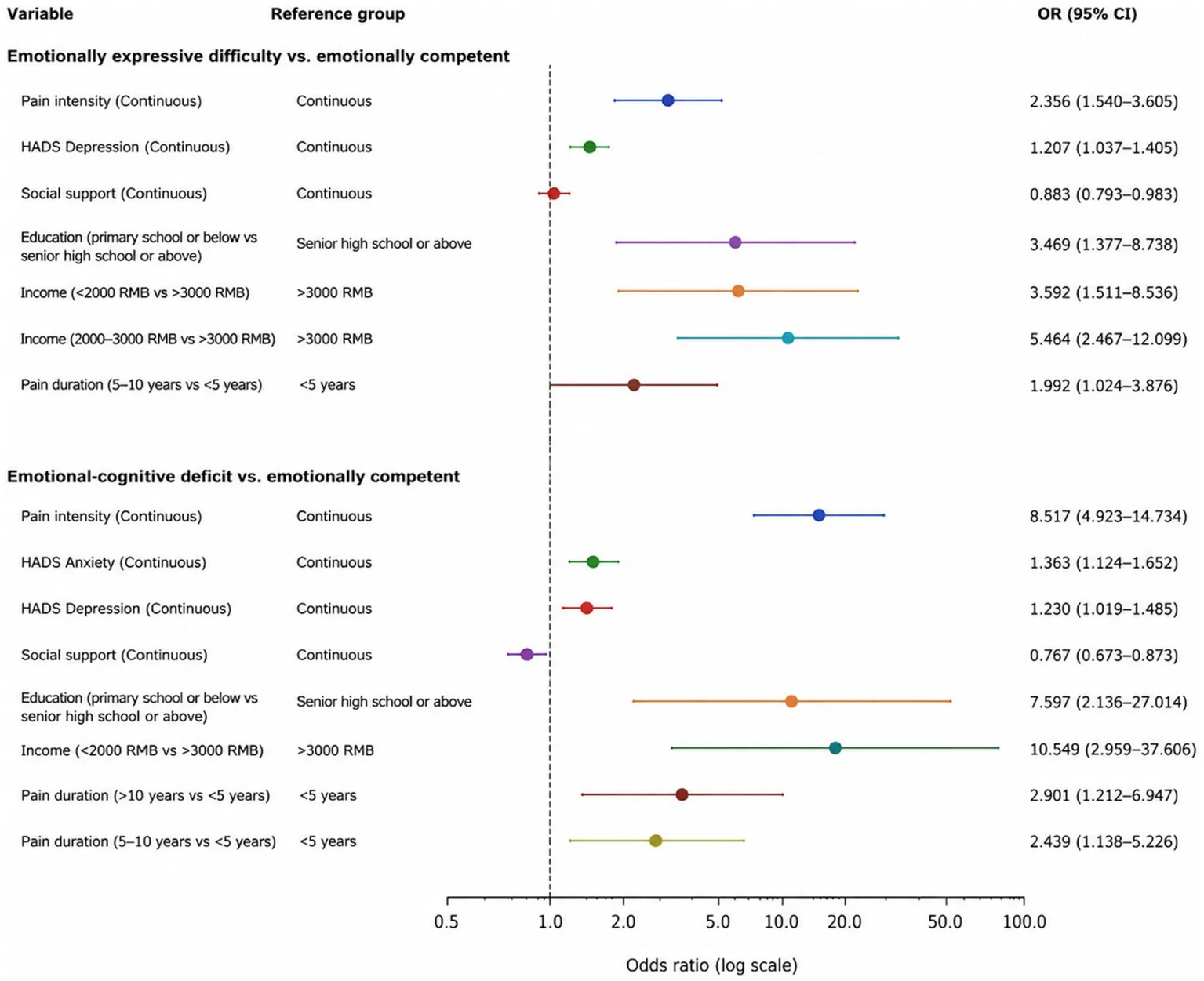
Adjusted associations between candidate predictors and alexithymia profile membership. Odds ratios and 95% confidence intervals were estimated using multinomial logistic regression, with the emotionally competent profile as the reference group. Values greater than 1 indicate higher odds of membership in the corresponding profile, whereas values less than 1 indicate lower odds. HADS, Hospital Anxiety and Depression Scale; RMB, renminbi.

A visual summary of the adjusted odds ratios and 95% confidence intervals is presented in Figure 3.

## 4 Discussion

This study identified three distinct alexithymia profiles among patients scheduled for TKA, indicating meaningful heterogeneity in the configuration and severity of alexithymic traits. Profile membership differed according to socioeconomic characteristics, pain burden, psychological distress, and perceived social support. These findings extend previous variable-centered research by demonstrating that patients with similar clinical diagnoses may differ substantially in their emotional awareness and communication-related characteristics. However, because of the cross-sectional design, the identified factors should be interpreted as correlates rather than determinants of profile membership.

The mean TAS-20 score in this sample was 57.02 ± 5.76, suggesting a moderate level of alexithymic traits. This finding was consistent with evidence that alexithymia is more common among people living with chronic pain than in the general population (8, 9). Elevated externally oriented thinking scores suggested that some patients might have relied more on external and somatic experiences when expressing distress, which is consistent with previous findings linking alexithymic traits with somatic symptom reporting and chronic pain experiences (31). Such a cognitive style may have been particularly important in TKA care because patients were required to communicate pain, fear, functional limitations, and rehabilitation difficulties to nurses and rehabilitation professionals.

The largest profile was the emotionally expressive difficulty profile. These patients appeared able to perceive emotional states but had difficulty describing them (32). In clinical practice, this profile might have been easily overlooked because patients might not have shown obvious psychological distress during routine assessment. However, expressive difficulties could still have interfered with pain reporting, rehabilitation feedback, and shared decision-making. Simple communication aids, emotion vocabulary cards, visual analog emotion scales, and structured nurse–patient conversations might have helped these patients verbalize perioperative concerns.

The emotional-cognitive deficit profile represented the most severe pattern, with high scores across all TAS-20 dimensions. This profile was associated with higher pain intensity, longer pain duration, greater anxiety and depression, lower social support, and socioeconomic disadvantage. The combination of intense pain and impaired emotional awareness might have amplified somatic attention and reduce the patient’s ability to engage in adaptive coping (33). Therefore, this group might have been considered for integrated interventions that combine pain management, psychological support, family involvement, and repeated communication checks, pending further longitudinal validation.

Pain intensity showed the strongest association with both alexithymia-related profiles, with the largest effect observed for the emotional-cognitive deficit profile. This finding was consistent with the hypothesis that severe pain might be associated with the allocation of attentional resources toward pain-related information and reduced emotional processing (8). Long pain duration was also associated with the emotional-cognitive deficit profile, suggesting that prolonged pain exposure might have contributed to emotional habituation, reduced interoceptive clarity, and more externally oriented coping. For nurses, this emphasized the importance of evaluating not only pain severity but also the patient’s ability to interpret and communicate pain-related emotional experiences.

Higher perceived social support was consistently associated with lower odds of profile membership. Supportive relationships were associated with interpersonal emotion regulation resources, which might have facilitated patients’ recognition, labeling, and communication of emotions (34). Family members could also have assisted with rehabilitation adherence and symptom monitoring (35). For patients with severe alexithymic traits, family-inclusive education might have been especially useful, provided that nurses guided families to use supportive rather than dismissive communication.

Anxiety was associated only with the emotional-cognitive deficit profile, whereas depression was associated with both higher-risk profiles. Anxiety has been associated with heightened bodily vigilance and catastrophic interpretations of pain, whereas depression has been linked to reduced motivation, difficulties in emotional clarity, and lower engagement with rehabilitation (36–38). These findings supported routine screening for anxiety and depression in patients with high TAS-20 scores and highlighted the potential value of referral pathways involving psychological or psychiatric support when clinically indicated.

Low educational attainment and low monthly income were the strongest predictors of membership in the emotional-cognitive deficit profile (39, 40). These factors might have been related to limited emotional vocabulary, access to health information, perceived control, and the capacity to seek psychological support. The large OR for low income should be interpreted cautiously because of the wide confidence interval, but the direction of association is clinically important. Nurses were encouraged to use plain language, teach-back methods, and individualized counseling for patients with limited educational or economic resources.

This study has several implications for pain management nursing. First, TAS-20 screening before surgery was associated with the identification of patients at risk for alexithymia-related communication barriers in this sample. Second, hypothetically, interventions could be tailored to profile characteristics: patients with expressive difficulty showed associations with potential benefits from emotion-labeling and communication training, whereas patients with emotional-cognitive deficits showed associations with needs for integrated pain-psychological management and family-inclusive support. These are theoretical considerations that require validation in interventional studies. Third, socioeconomic vulnerability was associated with profile membership, suggesting that nurses might consider this factor when planning perioperative education and rehabilitation guidance. Fourth, psychological distress and social support were associated with profile membership alongside pain intensity, supporting the potential value of comprehensive assessment for future research on individualized care planning.

### 4.1 Limitations

Several limitations should be acknowledged. First, the single-center cross-sectional design and convenience sampling in a tertiary hospital in northwestern China may limit generalizability to other regions, healthcare settings, or cultural contexts. Second, causal relationships between alexithymia profiles and associated factors cannot be established; the temporal stability of profiles and directionality of associations require longitudinal investigation. Third, all psychological variables were self-reported, which may be subject to recall or social desirability bias; although common method variance was unlikely to have substantially affected the findings, residual measurement bias cannot be completely excluded. Fourth, relatively wide confidence intervals for some socioeconomic variables indicated limited estimation precision in certain subgroup comparisons, warranting cautious interpretation. Finally, the study focused on preoperative psychological characteristics without assessing postoperative outcomes such as pain relief, functional recovery, rehabilitation adherence, or quality of life; the predictive value of profile-based assessment for postoperative recovery remains to be determined in prospective studies.

## 5 Conclusion

This study identified three distinct alexithymia profiles among patients scheduled for TKA, indicating meaningful heterogeneity in alexithymic traits within this population. Profile membership was associated with educational attainment, monthly income, pain intensity, pain duration, anxiety, depression, and perceived social support. These findings support further investigation of profile-informed preoperative assessment and nursing care. Prospective studies are needed to determine the stability of these profiles and whether profile-specific interventions can improve perioperative communication, psychological well-being, rehabilitation engagement, or postoperative outcomes.

## Data Availability

The raw data supporting the conclusions of this article will be made available by the authors, without undue reservation.
